# Microperimetry in hydroxychloroquine macular toxicity

**DOI:** 10.22336/rjo.2021.47

**Published:** 2021

**Authors:** Oswaldo Esteban Durán-Carrasco, Ruymán Rodríguez-Gil, Nicolás Pérez-Llombet-Quintana, Consuelo Fernández-Núñez, María Alberto-Pestano, Marta Alonso-Plasencia, Rodrigo Abreu-González

**Affiliations:** * Ophthalmology Department, Nuestra Señora de la Candelaria University Hospital, Spain

**Keywords:** microperimetry, mesopic, scotopic, retinal sensitivity

## Abstract

**Objective:** The aim was to evaluate the value of microperimetry (MP) in the early detection of toxic maculopathy caused by HCQ treatment in patients with normal fundoscopy, as well as normal structural optical coherence tomography (OCT).

**Materials and methods:** Microperimetry was performed in 13 patients under hydroxychloroquine treatment, who did not present fundoscopic or structural OCT alterations compatible with maculopathy. We used Nidek MP3s equipment (Nidek, Gamagori, Japan) with a 13-point pattern centered in fovea, in mesopic mode and in scotopic mode.

**Results:** The mean retinal sensitivity (MRS) in the study group was 27.25 +/ - 2.80 dB (95% CI 26.09 to 28.41 dB) while in the group of healthy volunteers 29.34 +/ - 2.18 dB (95% CI 28.67 to 30.1 dB). In scotopic mode, the mean sensitivity was 13.38 +/ - 1.43 dB (95% CI 12.79 to 13.97 dB) for HCQ users and 14.40 +/ - 2.1 dB (95% CI 13.76 to 15.04 dB) in the non-user group.

Central retinal sensitivity (CRS) was also lower in patients using HCQ 26.52 +/ -4.0 dB (95% CI 24.8 to 28.15 dB) vs. 29.06 +/ - 2.5 dB (95% CI 28.33 to 29.87 dB) in the control group in mesopic mode. The trend was repeated in scotopic CRS (10.85 +/ -1.84 dB vs. 12.16 +/ - 2.61 dB respectively).

**Discussion:** Our results showed that MP, especially in its mesopic mode, is a useful method to detect retinal toxicity caused by HCQ consumption in patients without funduscopic alteration and with normal macular OCT.

**Conclusions:** In mesopic mode, MRS was significantly lower in patients with long-term hydroxychloroquine treatment compared to those who did not use it, even in cases in which no fundoscopic or structural OCT alteration was detected.

**Abbreviations:** HCQ = hydroxychloroquine, MP = microperimetry, OCT = optical coherence tomography, BCVA = best corrected visual acuity, CRS = central retinal sensitivity, RS = retinal sensitivity, GEE = generalized estimating equations, MRS = mean retinal sensitivity, MfERG = multifocal electroretinogram, AMD = age-related macular degeneration

## Introduction

Chloroquine and hydroxychloroquine (HCQ) are derivatives of quinine initially used in the treatment of malaria, in addition to being used in the treatment of various rheumatologic and dermatologic pathologies [**1**,**2**]. The incidence of retinal toxicity is directly related to the dose and time of use of antimalarials. Thus, retinal toxicity occurs in 2% of cases in 10 years of treatment, and in up to 20% of cases after 20 years of continuous use of antimalarials [**3**,**4**]. 

Microperimetry (MP) is a diagnostic test that measures retinal sensitivity while simultaneously performing a fundoscopy, providing information on functional losses impairment and relating them to anatomical structural alterations; this type of examination can also be performed mesopically or scotopically [**5**-**7**]. 

The aim of our work was to determine whether microperimetry allows an earlier detection of retinal functional loss in patients treated with HCQ, measuring retinal sensitivity by mesopic and scotopic microperimetry in patients who have received treatment with HCQ, without signs of retinal toxicity in funduscopic examination or structural optical coherence tomography (OCT), the scanning methods commonly used in the follow-up of these patients in our service. 

## Materials and methods 

A cross-sectional study was performed between January 01, 2018 and December 31, 2019. Subjects were included from patients under HCQ intake for more than 12 months, who attended our hospital to undergo an ophthalmology exam and after being informed and signed the written informed consent. The study protocol was approved by the Institutional review board of Nuestra Señora de Candelaria University Hospital and followed the tenets of the Declaration of Helsinki. The inclusion criteria were lack of retinal toxicity signs by fundoscopy or macular OCT, maintaining a best corrected visual acuity (BCVA) of ≥ 0.8 on decimal scale and a spherical equivalent < +/ - 5 diopters. We then selected a group of healthy volunteers with a similar age and sex distribution. Both groups excluded diabetic patients and those diagnosed with macular dystrophies or identifiable ophthalmologic pathologies.

**Fig. 1:**
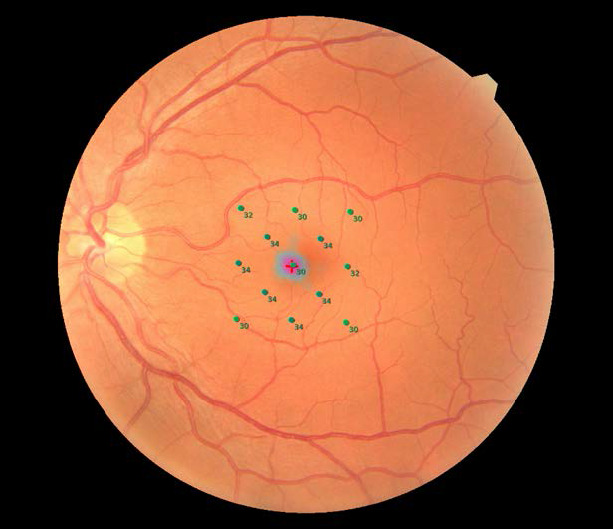
Thirteen-point grid used in our work and performed with the Nidek MP3S equipment (Nidek, Gamagori, Japan)

After a complete ophthalmological examination, subjects underwent a macular OCT centered on fovea using the Nidek RS-3000 Advance (Nidek, Gamagori, Japan) and a mesopic microperimetry using the Nidek MP3S (Nidek, Gamagori, Japan) without pharmacological mydriasis. We performed a self-designed protocol of 13 stimulus in a central area of 8º (**Fig. 1**). The size of the stimulus was Goldman III, the duration was 200 ms with a maximum sensitivity of 34 dB with a 4-2 strategy. Before starting scotopic we kept the patient in an environment of maximum darkness for 15 minutes, and then performed the MP in this mode, using the same grid, only modifying the parameters to Goldman V, and applying a maximum sensitivity of 24 dB, adjustments that were necessary to perform this scanning mode. The automatic eye position tracking system was always activated and it is worth mentioning that each MP was routinely started with a fixation test and a brief adaptation and practice period before the test.

All the patients had to present a normal macular structural OCT, nevertheless we collected central macular thickness after this examination in both groups. From the MP we took 13 mesopic retinal sensitivity points individually and then we combined the 5 central points (10,11,5,12,13), to estimate the central retinal sensitivity (CRS). Finally, an average value from all the retinal sensitivity points was made (mean retinal sensitivity). These values were also collected in scotopic mode. 

Data such as sex, age, treatment time, weight, dose, BCVA, were collected in the digital medical record.

*Statistical analysis*

**Fig. 2:**
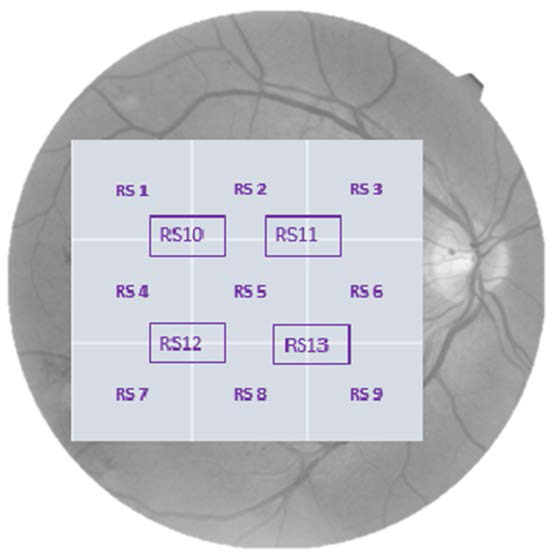
Diagram of studied points of retinal sensitivity in the right and left eye, respectively

Prior to the collection of retinal sensitivity, we developed a method for numbering each area corresponding to each point of the grid used (**Fig. 2**). Subsequently, using SPSS version 25 (IBM, Armonk, NY, USA), we processed the data using means and standard deviation for the variables related to the demographic data characterizing the sample.

To compare the results obtained in retinal sensitivity (RS), we initially applied T-Student, then the reliability of each diagnostic test was determined using the intraclass correlation coefficient test and Cronbach’s alpha index. Finally, due the size of the sample and the use of both eyes of a single individual (paired eyes) for the study in some cases, a generalized estimating equations (GEE) model was applied in order to enhance the statistical analysis and increase the statistical inference of the study.

## Results

Twenty-five eyes of 13 patients under treatment with HCQ and 41 healthy eyes in the control group corresponding to 21 healthy volunteers were evaluated. In the study group, 96% of the patients were women undergoing rheumatologic treatment. A mean age of 53.52 +/ - 9.41 years was found in HCQ-using patients, and 52.93 +/ - 12.75 years in healthy volunteers (p=0.841). 

The patients’ time of chloroquine use was 6.04 +/ - 4.7 years on average, with a mean dose of 280 +/ - 70 mg (95% CI 140 - 420) per day. The central macular thickness average in the group of HCQ treated subjects was 261.28 +/ - 15.13 µm (95% CI 255.34 - 267.84) and in the healthy group 261.59 +/ - 17.79 µm (95% CI 256.14 - 267.04) with no statistically significant difference (p= 0.941), (**Table 1**). 

**Tabel 1:** Age and macular thickness, study, and control group Patients on treatment with hydroxychloroquine

	N	Media	Standard deviation
Healthy age (years)	21	52,93	12,75
Age of HCQ users (years)	13	53,52	9,412
Macular Thickness Value in OCT, Healthy (microns)	21	261,59	17,79
Macular Thickness value in OCT, HCQ users (microns)	13	261,28	15,134
Time of HCQ use (years)	13	6,04	4,7
HCQ = Hydroxychloroquine; OCT = Optical Coherence Tomography			

In the mesopic MP, we obtained a mean retinal sensitivity (MRS) of 27.25 +/ - 2.80 dB (95% CI 26.09 - 28.41 dB) in the study group, while in the control group, the MRS was 29.34 +/ - 2.18 dB (95% CI 28.67 - 30.01 dB). Continuing with the analysis of the mesopic MP, when analyzing the thirteen areas individually, we found lower results (p < 0.05) in patients using HCQ in 9 of the analyzed points (except for areas 3, 4, 9 and 13). We found a mean SRC in individuals treated with HCQ of 26.50 +/ - 4.0 dB, (95% CI 24.8 - 28.15 dB) lower with respect to the control group (CRS= 29.10 +/ - 2.5 dB, 95% CI 28.33 - 29.87 dB). Both CRS and MRS were significantly lower (p = 0.001) in patients treated with HCQ.

In the scotopic mode, the MRS found in the study group was 13.38 +/ - 1.43 dB (CI 95% 12.79 - 13.97 dB) and 14.40 +/ - 2.1 dB (95% CI 13.76 - 15.04 dB) in the control group, being lower in the group of patients treated with HCQ, although when analyzing the areas individually in the scotopic MP we only found statistical significance (p< 0.05) when comparing area 7 with the healthy eyes group. Analyzing the central area, the mean CRS was 10.85 +/ - 1.84 dB (95% CI 10.10 - 11.62 dB) and 12.16 +/ - 2.61 dB (95% CI 11.36 - 12.96 dB) for the group of patients using HCQ and the control group respectively, showing significant results (p = 0.02) in both MRS and SC (**Table 2**, **3**). 

**Tabel 2:** Results of mesopic microperimetry at each of the points analyzed, central retinal sensitivity (CRS) and mean retinal sensitivity (MRS) obtained in the hydroxychloroquine users’ group and in the control group

Mesopic	Healthy media	Hydroxychloroquine media	95% confidence interval	P Value
Area 1 (dB)	29,86	28,56	-0,167 – 3,211	0,048
Area 2 (dB)	29,44	28,20	1,149 – 5,485	0,044
Area 3 (dB)	29,12	27,20	0,849 – 4,320	0,071
Area 4 (dB)	30,35	27,40	0,776 – 3,394	0,058
Area 5 (dB)	30,44	28,92	1,270 – 6,983	0,008
Area 6 (dB)	30,84	27,52	0,923 – 3,862	0,011
Area 7 (dB)	24,26	22,52	0,172 – 3,948	0,006
Area 8 (dB)	30,60	28,52	0,087 – 2,470	0,024
Area 9 (dB)	30,05	25,92	-,060 – 2,562	0,077
Area 10 (dB)	30,23	27,84	0,8706 – 3,310	0,066
Area 11 (dB)	29,14	27,08	0,9552 – 4,135	0,003
Area 12 (dB)	28,56	27,28	-0,167 – 3,211	0,019
Area 13 (dB)	28,65	27,40	1,149 – 5,485	0,185
SRM (dB)	29,34	27,25	-0,849 – 4,320	0,008
CRS (dB)	29,06	26,52	0,776 – 3,394	0,002

**Tabel 3:** Results of scotopic microperimetry at each of the analyzed points, central retinal sensitivity (CRS) and mean retinal sensitivity (MRS) obtained in the hydroxychloroquine users’ group and in the control group

Scotopic	Healthy media	Hydroxychloroquine media	95% confidence interval	P Value
Area 1 (dB)	16,56	16,64	-1,087 – 0,923	0,871
Area 2 (dB)	16,05	14,88	-0,101 – 2,434	0,071
Area 3 (dB)	16,74	15,92	-0,355 – 2,003	0,168
Area 4 (dB)	13,12	12,16	-0,610 – 2,522	0,227
Area 5 (dB)	13,72	12,80	-0,687 – 2,529	0,257
Area 6 (dB)	14,60	13,92	-0,899 – 2,268	0,391
Area 7 (dB)	8,09	5,44	0,473 – 4,833	0,018
Area 8 (dB)	15,21	14,00	-0,112 – 2,531	0,072
Area 9 (dB)	12,49	11,96	-1,514 – 2,571	0,607
Area 10 (dB)	13,40	11,92	-0,299 – 3,250	0,102
Area 11 (dB)	16,19	15,36	-0,314 – 1,966	0,153
Area 12 (dB)	14,84	13,72	-0,408 – 2,642	0,148
Area 13 (dB)	16,23	15,28	-0,572 – 2,478	0,217
SRM (dB)	14,40	13,38	0,070 – 1,965	0,036
CRS (dB)	12,16	10,85	0,121 – 2,492	0,031

Comparing the mesopic test with the scotopic test, the first of these microperimetry modes showed a Cronbach’s Alpha coefficient of 0.872, which translated as “good reliability”, while in the scotopic mode, the reliability was only “acceptable” (0.760). With this type of statistical analysis, it was possible to identify that in the mesopic MP areas 4 and 9 were the ones that contributed the least to the level of reliability of the test, and in the scotopic variant, the two areas that contributed the least to its reliability were areas 4 and 13. However, by suppressing them, the level of reliability of the tests did not increase.

After applying a generalized estimating equations (GEE) model, we obtained significant differences when comparing CRS and MRS in mesopic mode between HCQ users and non-users (p = 0.035, CI: 0.59-0.98 and p = 0.045 CI: 0.43 - 0.99 respectively). On the contrary, in scotopic mode, we did not obtain relevant results (**Table 4**). 

**Tabel 4:** Results of thickness, central retinal sensitivity (CRS) and mean retinal sensitivity (MRS) in the generalized estimated equations statistical model, considering the effect of using the two eyes of the same patient as an intra-subject variable Generalized estimating equations model

			Confidence Interval	
	Sig. (P)	Media	Superior	Inferior
Intercept	0,152	4796,173	0,044	524722367
Central macular thickness	0,964	0,999	0,962	1,037
Mesopic CRS	0,035	0,761	0,59	0,981
Scotopic CRS	0,637	0,913	0,627	1,331
Mesopic MRS	0,045	0,657	0,435	0,991
Scotopic MRS	0,968	1,01	0,617	1,652

## Discussion

Our results showed that MP, especially in its mesopic mode, is a useful method to detect retinal toxicity caused by HCQ consumption in patients without funduscopic alteration and with normal macular OCT. In addition to the preserved morphology of the foveal profile observed in all the patients before being included in the study, if we compared the central macular thickness measured by OCT in our sample (mean: 261.28 +/ - 15.13 µm) with the results obtained in other studies such as that of Solé et al., close values were evident (mean: 261.31 +/ -17.67 µm) despite having used different devices. We can state that these macular thickness values are similar to those obtained in studies that analyzed this value in a healthy population [**8**]. One of the weaknesses of our work in this aspect has been the use of central macular thickness, since it has been seen in studies such as that of Seong et al. [**9**], which is the pericentral zone that is affected earlier in subjects undergoing treatment with HCQ. 

The American Academy of Ophthalmology recommends screening for macular toxicity due to HCQ by means of automated campimetry and spectral domain optical coherence tomography (OCT), with the possibility of extending the exploration with multifocal electroretinogram (MfERG) if it is necessary. Control usually begins after five years of HCQ consumption at recommended doses (5 mg/ kg/ day) [**10**,**11**]. However, there are studies that show a decrease in retinal sensitivity by MP in patients treated with CQ or HCQ earlier with doses within the recommended limits [**12**,**13**]. 

Our control group presented a normal value of mean retinal sensitivity (29.49, SE = 0.32 dB) when compared with other works found in literature, using the same device as the one used in our study (Nidek, Gamagori, Japan). Balasubramania et al. [**14**] have found a mean of 25.02 dB (+/ -1.06). However, in a large group of 237 healthy volunteers, Molina Martín et al. [**15**] found a mean value of 32.90 dB (interquartile range 1.80 dB) using MAIA equipment (Centervue, Padova, Italy), both results being comparable with those obtained in our control group.

The retinal sensitivity obtained in mesopic MP in patients using HCQ was lower than in the group of healthy volunteers (26.75 vs. 29.49 dB), and this difference has been found in several works published in literature. Jivrajka et al. [**16**] found an SRM in the central 10° retina of 14.7 (±1.9 dB) in their study group compared to 16.5 (±2.1 dB) in controls using OPKO Spectral OCT/ SLO equipment (Miami, Florida, USA). Similarly, Eren M et al. [**17**] detected a mean retinal sensitivity of 84 (63-100) dB versus SRM 89.7 (83-98) dB in their group of healthy volunteers (Nidek MP 1, Technologies, Albingasego, Italy). Values more similar to ours were found by Molina et al. [**18**], with a mean of 29.05 +/ - 0.57 dB versus 26.05 +/ - 2.75 dB. These researchers gathered a group of 14 patients (28 eyes) in treatment with HCQ. However, in a sample of 194 users of this type of drugs, the research of Martinez Costa et al. [**19**] found an SRM of 26.83+/ -2.27 dB. The MAIA equipment was used (Centervue, Padova, Italy) in both cases. It is worth mentioning that these researchers, as us, found a statistically significant decrease (p<0.05) of the central retinal sensitivity in the group of patients in treatment with antimalarials. Another important aspect to consider was the difference in the device used in each study to perform the MP, which influenced the values obtained, showing how little comparable they were.

However, in patients with age-related macular degeneration (AMD), there are studies that have even shown superiority of scotopic versus mesopic MP in the detection of retinal sensitivity loss in early stages [**20**,**21**]. This trend has not been reflected in our study, as we have observed better reliability in mesopic scanning for detecting alterations in retinal sensitivity in patients under treatment with HCQ, which may have been related to the lower number of rods at the macular and foveal area [**22**,**23**].

Our research had certain limitations such as the lack of comparison with the test considered the “gold standard”, the multifocal electroretinogram (mfERG), as this exploration would have provided us with data on the functional state of the retina. One of the most important statistical limitations usually observed in the studies performed in our specialty is the use of the two eyes of a single individual because it can be assumed that they will both show similar characteristics. However, a decrease in the statistical power of the works that use the right eyes or the left eyes has also been exclusively seen. This situation, as well as the reduced sample size, have tried to be solved by applying the GEE model. This statistical tool is the extension of a linear regression within a longitudinal framework where repeated measurements are performed in everyone [**24**-**26**].

The use of the scotopic modality of MP to analyze retinal sensitivity in this group of patients is novel, and although the results are not encouraging, the use of this type of test in this kind of patients should continue to be studied in larger populations.

## Conclusions

With our study, we can conclude that MRS in patients using hydroxychloroquine is significantly lower than in patients who do not use it, especially in mesopic microperimetry. In our research, we found that the diagnostic reliability is higher in mesopic MP than in scotopic MP to detect decreased retinal sensitivity in patients under treatment with HCQ and without maculopathy clinically evident or by OCT. We believe that microperimetry may be useful in screening for HCQ-induced retinal toxicity.

**Conflict of Interest statement**

The authors state no conflict of interest.

**Informed Consent and Human and Animal Rights statement**

Informed consent has been obtained from all individuals included in this study.

**Authorization for the use of human subjects**

Ethical approval: The research related to human use complies with all the relevant national regulations, institutional policies, is in accordance with the tenets of the Helsinki Declaration, and has been approved by the review board of Nuestra Señora de la Candelaria University Hospital, Canarias, Spain.

**Acknowledgements**

None.

**Sources of Funding**

None.

**Disclosures**

None.
